# The Anti-Inflammatory Effect and Mucosal Barrier Protection of *Clostridium butyricum* RH2 in Ceftriaxone-Induced Intestinal Dysbacteriosis

**DOI:** 10.3389/fcimb.2021.647048

**Published:** 2021-03-25

**Authors:** Yuyuan Li, Man Liu, He Liu, Xue Sui, Yinhui Liu, Xiaoqing Wei, Chunzheng Liu, Yiqin Cheng, Weikang Ye, Binbin Gao, Xin Wang, Qiao Lu, Hao Cheng, Lu Zhang, Jieli Yuan, Ming Li

**Affiliations:** ^1^ Advanced Institute for Medical Sciences, Dalian Medical University, Dalian, China; ^2^ College of Basic Medicine, Chengdu University of Traditional Chinese Medicine, Chengdu, China; ^3^ College of Basic Medical Science, Dalian Medical University, Dalian, China; ^4^ Marketing Department, Hangzhou Grand Biologic Pharmaceutical Inc., Hangzhou, China

**Keywords:** *****Clostridium butyricum*, CB RH2 in Intestinal Dysbiosis, immune response, mucosal barrier function, gut microbiota

## Abstract

This study aimed at determining the beneficial effect of *Clostridium butyricum* (CB) RH2 on ceftriaxone-induced dysbacteriosis. To this purpose, BALB/c mice were exposed to ceftriaxone (400 mg/ml) or not (control) for 7 days, and administered a daily oral gavage of low-, and high-dose CB RH2 (10^8^ and 10^10^ CFU/ml, respectively) for 2 weeks. CB RH2 altered the diversity of gut microbiota, changed the composition of gut microbiota in phylum and genus level, decreased the F/B ratio, and decreased the pro-inflammatory bacteria (*Deferribacteres*, *Oscillibacter*, *Desulfovibrio*, *Mucispirillum* and *Parabacteroides*) in ceftriaxone-treated mice. Additionally, CB RH2 improved colonic architecture and intestinal integrity by improving the mucous layer and the tight junction barrier. Furthermore, CB RH2 also mitigated intestinal inflammation through decreasing proinflammatory factors (TNF-α and COX-2) and increasing anti-inflammatory factors (IL-10). CB RH2 had direct effects on the expansion of CD4^+^ T cells in Peyer’s patches (PPs) *in vitro*, which in turn affected their immune response upon challenge with ceftriaxone. All these data suggested that CB RH2 possessed the ability to modulate the intestinal mucosal and systemic immune system in limiting intestinal alterations to relieve ceftriaxone-induced dysbacteriosis.

## Introduction

The intestine is different from the other organs of the human body because it consists of a physical and immunological protective barrier against foreign antigens and pathogens (Curciarello et al., 2019; Liang et al., 2020). Any dysfunction of intestinal barrier may promote and sustain an inflammation of the intestine (Miner-Williams and Moughan, 2016). The gut microbiota has diverse effects on the regulation of many various physiological processes, including nutrient digestion and acquisition, modulation of the gut-specific immune system, and protection from infectious pathogens (Wang et al., 2019). Recently, growing evidences have demonstrated that the interplay between the gut microbiota, the intestinal barrier and the mucosal immune system is profoundly altered in multifarious diseases, such as cardiovascular disease (Lewis and Taylor, 2020), infectious disease (Rosel-Pech et al., 2020), inflammatory bowel disease (Lee et al., 2020), autoimmune disease (Antonini et al., 2019), and a variety of cancers (Yoo et al., 2020).

Antibiotics, a mainstay of modern medicine, have saved many thousands of lives from infectious disorders since their widespread introduction. Unfortunately, the overuse of antibiotics, in particular, β-lactams, has led to intestinal microbiota imbalance, which considerably weaken colonization resistance and result in pathobiont overgrowth (Burdet et al., 2019; Venturini et al., 2021). Ceftriaxone, a β-lactam antibiotic, has high biliary elimination, which may result in a pronounced impact on the intestinal microbiota (Burdet et al., 2019). Ceftriaxone-induced intestinal dysbacteriosis is a growing health concern and a focus of research (Gao et al., 2012; Lama et al., 2019). It was shown that ceftriaxone-induced dysbiosis impacted the integrity of mucosal epithelial layer, which were accompanied by overexpression of mucin-2 (MUC-2) and overproduction of defensins and inflammatory cytokines (Li et al., 2015).

Probiotics supplement is an important way to modulate systemic and mucosal immune function, improve intestinal barrier function, and alter gut microecology. *Clostridium butyricum* (CB) is a strictly anaerobic gram-positive and endospore-forming probiotic with acid and heat resistant properties (Liu et al., 2020a), which has been widely used for improving gastrointestinal function. Furthermore, CB commonly exists in the gut of normal healthy individuals (humans and animals), which produces acetic and butyric acid, two important components of short chain fatty acids (SCFAs) synthetized by gut microbiota (Detman et al., 2019). Butyrate is known as a primary energy source for colonocytes and functional substances for alleviating colitis (Han et al., 2020). Qiao et al. (2015) observed that butyrate significantly attenuated intestinal ischemia and reperfusion injury *via* preservation of intestinal tight junction (TJ) barrier function and suppression of inflammatory cell infiltration in the intestinal mucosa. Supplementation with CB has been shown to improve glycemic indexes and normalize blood lipids and inflammatory tone, showing an anti-diabetic effect of CB (Doumatey et al., 2020). CB also plays an important role in providing nutrients for the host and maintaining the balance of the microbial ecosystem in the intestine, while exhibiting a high tolerance to several antibiotics (CB strain: UCN-12; Chen et al., 2018). Therefore, CB has been used to treat several gastrointestinal diseases in clinical, such as diarrhea (Guo et al., 2019) and inflammatory bowel disease (IBD) (Bin et al., 2016) and irritable bowel syndrome (IBS) (Sun et al., 2018). Beyond its protective effects in the digestive tract, CB may have a positive impact on therapeutic efficacy of immune checkpoint blockade in patients with lung cancer, which was impaired by antibiotics-induced dysbiosis (Tomita et al., 2020). It was reported that CBM 588 had contributed to the gut epithelial barrier protection and anti-inflammatory effect in clindamycin-induced dysbiosis (Ariyoshi et al., 2020, Hagihara et al., 2020). However, there are few reports on the potential protect functions of CB in regulating gut microbiota and maintaining intestinal barrier function in ceftriaxone-induced dysbiosis.

In this study, we aimed to explore the mechanism by which CB RH2 intervention could improve the intestinal barrier function and alleviate ceftriaxone-induced dysbiosis. We hope our study can help to understand the capabilities of CB RH2 to enhance the T cells immune response and restore gut epithelial barrier function, which provides a theoretical support for the development of CB RH2 as a functional food ingredient.

## Materials and Methods

### Animals

All experiments were performed using protocols approved by the committee for animal care and use at Dalian Medical University (SYXK [Liao] 2018-0002). Male BALB/C mice (aged 6–8 weeks, weighing 18–22 g) were gained from the Experimental Animal House of Dalian Medical University, where they were maintained under stress-free and specific pathogen-free conditions (light/dark cycles of 12 h) at a room temperature of 24 ± 1 °C and 65 ± 15% humidity. All mice had free access to food and sterile water.

### Bacterial Strain

Freeze-dried bacteria powder of *C. butyricum* RH2 containing 2.4 × 10^10^ CFU/g was obtained from Grand Biologic Pharmaceutical (Chongqing) INC. It was cultured anaerobically with Man, Rogosa, and Sharpe (MRS) broth (Merck, Darmstadt, Germany) at 37°C. The growth of the culture was monitored by reading the optical density (OD) at 600 nm. The concentration of the bacteria was adjusted to 10^10^ and 10^8^ colony forming units (CFU)/ml.

### Induction of Intestinal Dysbacteriosis and Experimental Design

A total of 32 BALB/C mice were randomly divided into four groups (n = 8 for each group): one group (CON group) was only provided with physiological saline, mice in CS group and 2 CB groups were administrated by 0.2 ml ceftriaxone sodium (400 mg/ml) intra-gastrically twice a day with an interval of 6 h for 7 days to establish the dysbiosis model. Mice were given 10^8^ CFU/ml (LCB group) and 10^10^ CFU/ml (HCB group) CB RH2, suspended in 0.2 ml physiological saline once daily for 14 days after CS administration.

## DNA Extraction, 16S rDNA Sequencing and Bioinformatics Analysis

Extraction of bacterial DNA from mice feces of four groups was performed using E.Z.N.A.^®^ Stool DNA Kit (Omega Bio-Tek, Norcross, GA, USA) following to the manufacturer’s guidelines. The DNA concentration was assessed using NanoDrop 2000 spectrophotometer (Thermo Scientific, Wilmington, USA). The universal target V3–V4 regions of the 16S rDNA gene were amplified *via* PCR using barcoded primers 515 F (5’-GTGYCAGCMGCCGCGGTAA-3’) and 806R (5’-GGACTACNVGGGTWTCTAA-3’). Amplicons were excised from 1.5% agarose gels and purified with the QIAquick Gel Extraction kit (Qiagen, Germany). Then they were sequenced and the data were analyzed on an Illumina HiSeq platform (Novogene Bioinformatics Technology Co., Ltd., Beijing, China) using a method described previously (Li et al., 2017). The Ribosomal Database Project (RDP) Classifier 2.8 was used for exploiting taxonomical information and belief assignment of all sequences at 50% confidence after the raw sequences were identified by their unique barcodes. Operational taxonomic units (OTUs) present in more than 50% of the fecal samples were identified as core OTUs. Community diversity (alpha diversity) was measured by observed species and Shannon index, while community richness was evaluated by Chao1 index. Beta diversity was examined using principal coordinate analysis (PCoA) with weighted UniFrac analysis in R software and Unweighted Pair-Group Method with Arithmetic mean clustering (UPGMA).

### Histologic Analysis of Colon

At 22th day, colon tissues were collected and fixed in 10% neutral buffered formalin, dehydrated, and paraffin-embedded. The colon sections were hematoxylin/eosin (HE) stained and analyzed by the same pathologist in a blinded manner to evaluate their morphological characteristics. Histopathology was quantified based on the scoring system, focusing on the following parameters: (1) destruction of normal epithelial architecture; (2) inflammatory infiltration; (3) edema of the mucosa; (4) vascular dilatation and congestion; (5) goblet cell loss and (6) crypt abscesses. The scores for each parameter were then combined with a maximal possible score of 14 according to the method in previous report (Li et al., 2015).

### Transmission Electron Microscopy

Fresh colon tissues were separated and fixed with 2.5% glutaraldehyde at room temperature for 2 h and 1% osmium tetroxide for another 2 h. Then the samples were dehydrated in ethanol, passed through propylene oxide, and embedded in Spurr resin. Ultra-thin sections (100 nm) were cut and stained with 4% uranyl acetate for 10 min and Reynold’s lead citrate for 1.5 min. Samples were observed under a transmission electron microscope (JEM-1400, Olympus, Japan) at an accelerating voltage of 80 kV.

### Detection of Cytokines

Colon tissues were homogenized in 0.9% physiological saline to obtain a 10% homogenate, and then centrifuged at 8,000×*g* for 20 min at 4°C. The supernatant was used to quantify defensins using ELISA kits (USCN, USA). Peripheral blood was collected from the heart and centrifuged immediately at 1,500×*g* for 15 min at 4°C to obtain serum and then stored at −80°C until analyses. The concentrations of serum lipopolysaccharides (LPS) and interleukin-10 (IL-10) were also detected using ELISA kits (USCN, USA) according to the manufacturer’s instructions.

## RNA Extraction and Real-Time Semi-Quantitative PCR

Total RNA in mouse colon tissues was extracted using TRIzol Reagent (Bio-Rad Laboratories). The complementary DNA (cDNA) was synthesized using the AffinityScript Multiple Temperature cDNA synthesis Kit (Stratagene, La Jolla, CA, USA). The reactions were carried out in 384-well plates on the QuantStudio 6 Flex real-time PCR system (Life Technologies) using ChamQ Universal SYBR qPCR Master Mix (Vazyme). The sequences of primers are designed by the Primer Premier 5.0 software and summarized in **Table 1**. The results were analyzed using the 2^−△△ct^ method.

**Table 1:** List of PCR primers and amplicon size.

Primers	Sequence (5’-3’)	Size (bp)
ZO-1	Forward: CATCATTCGCCTTCATAC Reverse: TTGCTTAGAGTCAGGGTTA	142 bp
Occludin	Forward: TTGAAAGTCCACCTCCTTACAGA Reverse: CCGGATAAAAAGAGTACGCTGG	129 bp
Claudin-1	Forward: TACTTTCCTGCTCCTGTCC Reverse: CTCTTCCTTTGCCTCTGTC	112 bp
Claudin-4	Forward: CCTTCATCGGCAGCAACA Reverse: GGCGAGCATCGAGTCGTA	116 bp
MUC-2	Forward: CGGGAAATGCTGTCCAGTTTAT Reverse: ACGTTGAGCTGGGTGCTGTT	150 bp
TNF-α	Forward: AGATCATCTTCTCAAAATTCGAGTG Reverse: TACAACCCATCGGCTGGC	281 bp
COX-2	Forward: AGAAGGAAATGGCTGCAGAA Reverse: GCTCGGCTTCCAGTATTGAG	194 bp
β-Actin	Forward: AGCCATGTACGTAGCCATCC Reverse: GCTGTGGTGGTGAAGCTGTA	222 bp

## Western Blot

Total protein samples were extracted from murine colon tissues. Equal amounts of protein were fractionated by sodium dodecylsulfate polyacrylamide gel electrophoresis (SDS-PAGE) and transferred onto polyvinylidene difluoride (PVDF) membranes. Following transfer, membranes were blocked with 5% (w/v) milk in TBST buffer for 1 h at room temperature and then incubated with primary antibodies overnight at 4°C. The following specific primary antibodies were used: Mouse anti-ZO-1 (dilution; 1:1,000, Invitrogen, Camarillo, CA, USA), mouse anti-Occludin (dilution; 1:1,000, Invitrogen, Camarillo, CA, USA), rabbit anti-Claudin 1 (dilution; 1:1,000, Abbkine, San Diego, CA, USA), rabbit anti-Claudin 4 (dilution; 1:1,000, Abbkine, San Diego, CA, USA), and rabbit anti-β-actin (dilution; 1:1,000, Proteintech Group, Rosemont, USA). After washing with TBST buffer, the secondary antibodies conjugated with horseradish peroxidase (HRP, USCN, USA) were used to show the bands. The target proteins were detected with WesternBright™ ECL substrate and images were captured by Imager-Bio-Rad (Bio-Rad Laboratories, Inc., Hercules, CA, USA). For the densitometric analysis, the band intensities were quantified using Image J software (NIH).

## Flow Cytometry

The fresh spleen and intestinal Peyer’s patches (PPs) of mice was removed and ground in PBS buffer to isolate cells. Cell suspensions (1 × 10^6^) were incubated with anti-mouse CD16/CD32 mAb to block Fcγ receptors for 60 min and then stained on ice with PE-labeled CD4 (H129.19) FITC-labeled CD8 (53-6.7) mAbs for 60 min. The mAbs used in this study are all purchased from BD Biosciences. Flow cytometric analysis was performed with the Accuri C6 flow cytometer (BD Bioscience, USA), and data were analyzed with Flow Plus 1.0.264.15 Software.

## Culture of T Cells *In Vitro*

In order to investigate the effects of CB RH2 on proliferation of T cells in the PPs of mice, total lymphocytes from the PPs of the control mice were isolated and incubated with different concentrations of CB RH2 supernatant and cell lysate, respectively. According to the culture method as described in our previous study (Zhou et al., 2020), 1 mL of the CB RH2 culture broth (OD_600_ = 1.5) was centrifuged at 2,500 rpm for 5 min, and the culture supernatant was regarded as bacterial secretion. Then the pellet was resuspended in MRS for ultrasonic crushing to release the intracellular components, followed by centrifuging for 5 min at 2,500 rpm. Different dilutions of the supernatant, which was regarded as CB RH2 lysate, were added to the single-cell suspension after filtration through a 0.2 µm-filter. The MRS medium was used as control.

## Statistical Analysis

All data were presented as arithmetic mean ± standard error of mean (SEM). Data sets that involved more than two groups were analyzed using one-way ANOVA followed by turkey’s test (compare all pairs of columns); when two groups were compared and data obey normal distribution and even variance, a student’s t test was performed with the assistance of GraphPad Prism Program (Version 7.04; GraphPad Software Inc., La Jolla, CA, USA). The significance for PCoA (beta-diversity) analyses, which was tested with multivariate permutation tests using the nonparametric method “Adonis” included in the package “vegan” of the QIIME-incorporated version of “R”. Results were considered statistically significant with a p-value of less than 0.05.

### Accession Number

The sequence data from this study are deposited in the GenBank Sequence Read Archive with the accession number RPJNA689675.

## Results

### CB RH2 Modulates the Gut Microbiome Composition Under Ceftriaxone-Induced Intestinal Dysbiosis

**Figure 1:**
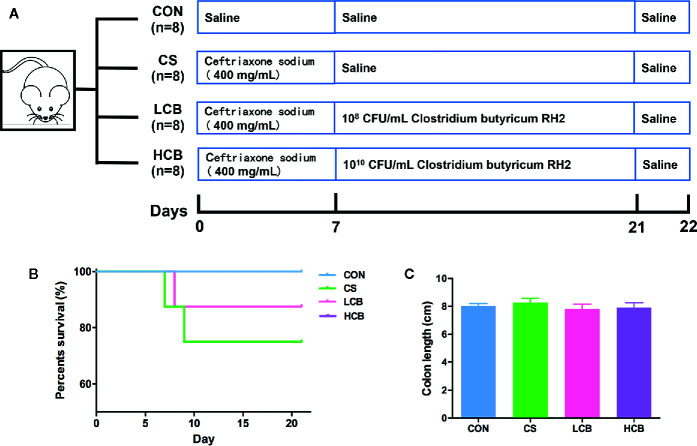
Schematic overview of ceftriaxone treatment and CB RH2 administration. **(A)** Experimental protocol for ceftriaxone treatment with administration of CB RH2 as intervention; **(B)** Survival rate; **(C)** Colon length (cm), data are represented as mean ± SEM of eight mice in each group.

**Figure 2:**
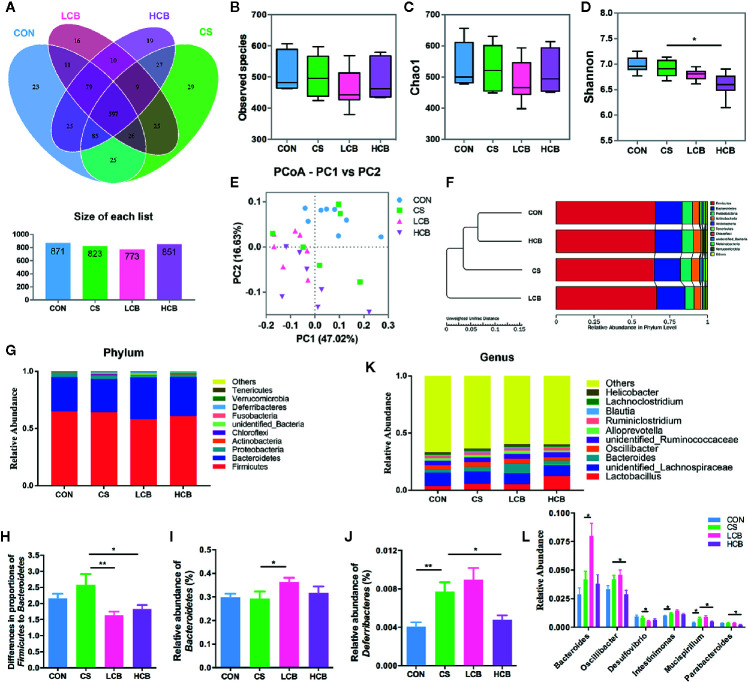
Evaluation of illumina HiSeq sequencing data showing that CB RH2 could modulate the structure and composition of gut microbiota. **(A)** Venn diagram of shared and independent bacterial OTUs in different experimental groups (n >6); **(B–D)** Analysis of α-diversity of gut microbiota by Observed species, Chao1 and Shannon index; **(E)** Principal coordinate analysis (PCoA) based on weighted Unifrac distances among different samples. PC1 and PC2 account for 63.65% of the variation; **(F)** Multivariate analysis of variance from PCoA matrix scores using UPGMA method based on weighted Unifrac distances; **(G)** The average abundance of microbial community in different mice groups at phylum level; **(H)** The ratio of *Firmicutes* to *Bacteroidetes*; **(I, J)** Statistical analysis for *Bacteroidetes* and *Deferribacteres* at the phylum level; **(K)** Bar charts at genus level of gut microbiota in the four groups; **(L)** Relative abundance of *Bacteroides*, *Oscillibacter*, *Desulfovibrio*, *Mucispirillum* and *Parabacteroides* are significantly manipulated by CB RH2 at genus level. Statistical analysis was performed using the t tests method. All values are mean ± SEM (n >6). *p < 0.05, **p < 0.01.

The experimental design is shown in **Figure 1A**. During the recovery after administration of ceftriaxone, there were two incidences of mortality in CS group, and only one incidence of mortality in each CB group (**Figure 1B**). It suggested that CB RH2 could significantly promote the recovery from ceftriaxone-induced dysbiosis. There were no significant changes in the length of colon (**Figure 1C**). To reveal the impact of CB RH2 on gut microbiota under ceftriaxone treatment conditions, we performed the analysis of the V3–V4 region of 16S rRNA gene sequences. Sequences were classified into 1,006 operational taxonomic units (OTUs) using a 97% similarity. The Venn diagram (**Figure 2A**) showed that there were 597 shared OTUs among four groups. The OTU number of the control group was 871, which was higher than that of the other groups. The unique OTUs of control, CS, LCB and HCB groups were 23, 29, 16 and 19 respectively. The observed species and Chao1 were used to estimate the community richness, while the Shannon index was used to evaluate the species diversity. The alpha diversity analysis results showed no significant differences between every two groups, except HCB group existed lower community diversity compare with CS group (P = 0.0123; **Figures 2B–D**). Additionally, the Bray-Curtis dissimilarities were calculated and displayed by PCoA and UPGMA (**Figures 2E, F**). Results showed a strong clustering of the gut microbiota composition for each group (control vs CS, R = 0.178, P = 0.002; CS vs LCB, R = 0.112, P = 0.141; CS vs HCB, R = 0.199, P = 0.001), which suggested that high concentration of CB RH2 made a certain impact on the gut microbiota composition of the ceftriaxone treated mice.

Alterations of the murine gut microbiota at the phylum level were shown in **Figure 2G**. We found that the ratio of *Firmicutes* to *Bacteroidetes* (F/B), an indicator of microbial imbalance (Mulder et al., 2020, Yang et al., 2020), was significantly lower in CB groups when compared with CS group (CS vs LCB, P = 0.0071; CS vs HCB, P = 0.0464; **Figure 2H**). The *Bacteroidetes* abundance was increased in LCB group (P = 0.05; **Figure 2I**), while the *Firmicutes* abundance was not changed significantly among the four groups (P = 0.174). Moreover, ceftriaxone treatment greatly increased the levels of *Deferribacteres* compared to the control group (P = 0.003). However, following high dose of CB RH2 supplementation, the proportions of *Deferribacteres* returned to control levels (P = 0.015; **Figure 2J**). When differences in the microbiota at the genus level were compared (**Figure 2K**) and the statistical differences were further tested (**Figure 2L**). The proportion of *Bacteroides* significantly increased following CB RH2 supplementation compared to the CS group (CS vs LCB, P = 0.014), while the proportion of *Oscillibacter* (CS vs HCB, P = 0.024), *Desulfovibrio* (CS vs LCB, P = 0.031), *Mucispirillum* (CS vs HCB, P = 0.028) and *Parabacteroides* (CS vs HCB, P = 0.019) significantly decreased. We also found that CB RH2 tended to decrease the proportion of *Intestinimonas*, which was enhanced by ceftriaxone treatment (P = 0.016). These results suggested that CB RH2 attenuated intestinal bacteria dysbiosis by regulating the composition of gut microbiota.

### Impact of CB RH2 on the Repair of Intestinal Barrier Integrity

**Figure 3:**
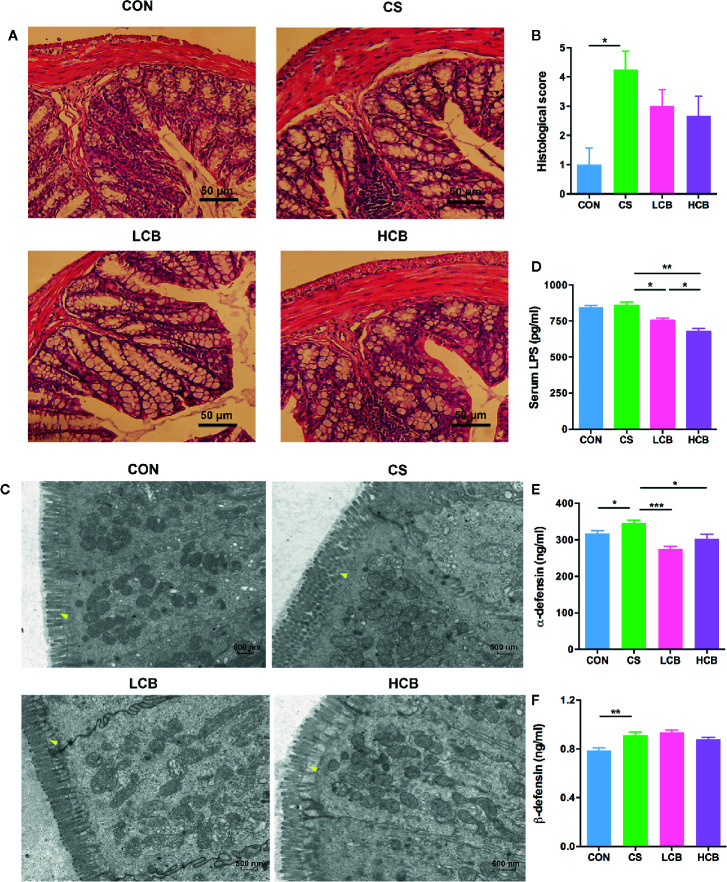
CB RH2 changes the mechanical barriers in intestinal mucosa of ceftriaxone-treated mice. **(A)** H&E-stained results for the sections of mouse colon; **(B)** Histopathological analysis of the H&E-stained sections; **(C)** Transmission electron microscopy (TEM) analysis of colon; **(D)** LPS of serum were detected by ELISA following the manufacturer’s protocol; **(E, F)** The concentrations of α-defensin and β-defensin in colon of mice. All data were evaluated as mean ± SEM (n = 5), *p <0.05, **p <0.01, ***p <0.001.

H&E-stained colorectal sections showed that the ceftriaxone-treated mice exhibited serious injuries. They had an increase in hyperplasia of the colonic mucosa, distorted tissue architecture, inflammatory infiltration, and more severe vascular dilatation and congestion than that of control mice. In contrast, CB RH2 administration improved the histological structure versus the CS group, albeit not significantly so (**Figure 3A**). The histological score of the colon in mice treated with ceftriaxone was higher compared with control and CB RH2 administrated mice (P = 0.018; **Figure 3B**). Although there was no significant difference, two CB groups showed numerically lower scores than that of the ceftriaxone administration group.

Transmission electron microscopy (TEM) results further confirmed the effect of CB RH2 on the protection of intestinal mechanical barrier. It could be seen very intuitively from **Figure 3C** that the microvilli in the colonic epithelium of the control group were compact and arranged neatly, forming a complete tight junction. Mice in CS group had disordered arrangement of microvilli and tight junction structure disruption. In contrast, CB RH2-treated mice demonstrated the villi arranged neatly. In addition, CB RH2 significantly decreased the serum level of lipopolysaccharide (LPS) in ceftriaxone-induced dysbiosis, which was the indication of gut permeability (CS vs LCB, P = 0.015; CS vs HCB, P = 0.0017; **Figure 3D**). Moreover, the effect of CB RH2 treatment in high dose was stronger than that of low dose (P = 0.027; **Figure 2D**).

The antimicrobial proteins (AMPs), such as α-defensins and β-defensins secreted by intestinal epithelia cells, were also detected. We found that total α-defensin (P = 0.032; **Figure 3E**) and β-defensin (P = 0.0048; **Figure 3F**) levels were higher in the CS group compared with the control group, which showed the consistent result with our previous *in vivo* study with ceftriaxone-induced dysbiosis (Li et al., 2015). Lower concentrations of total α-defensin were detected in mice treated with CB RH2 (CS vs LCB, P = 0.0002; CS vs HCB, P = 0.016; **Figure 3E**). The level of total β-defensin in the HCB group decreased gradually, but no significant differences were detected compared with the CS group.

### CB RH2 Modulates Expression of Tight Junction Proteins and MUC-2 in the Colon of Mice Treated With Ceftriaxone

**Figure 4:**
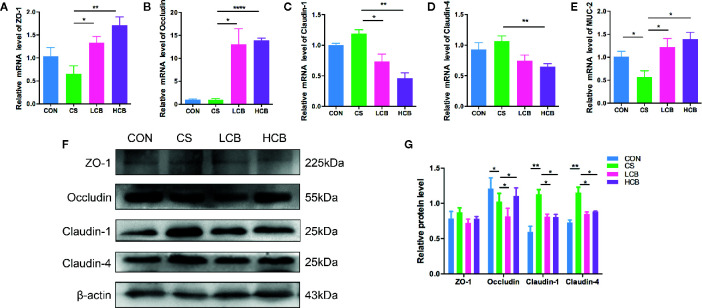
CB RH2 enhances intestinal barrier function of ceftriaxone-treated mice. **(A–E)** The relative RNA expression of genes encoding zonula occludens (ZO-1), Occludin, Claudin-1, Claudin-4, and mucin-2 (MUC-2) in colon tissues of mice, detected by qPCR; **(F, G)** Representative blots and comparison of protein expression of ZO-1, Occludin, Claudin-1, Claudin-4, and MUC-2 by western blot with β-actin as internal control. All data were evaluated as mean ± SEM (n = 5), *p < 0.05, **p < 0.01, ****p < 0.0001.

To further characterize the protective effects of CB RH2 on the epithelial layer after CS administration in mice, the mRNA and protein expression levels of the tight junction (TJ)-related proteins, ZO-1, Occludin, Claudin-1 and Claudin-4 were assessed using real-time PCR and western blot analysis, respectively. The mRNA expression levels of ZO-1 (CS vs LCB, P = 0.0253; CS vs HCB, P = 0.0065; **Figure 4A**) and Occludin (CS vs LCB, P = 0.0142; CS vs HCB, P <0.0001; **Figure 4B**) were increased, and those of Claudin-1 (CS vs LCB, P = 0.0218; CS vs HCB, P = 0.0017; **Figure 4C**) and Claudin-4 (CS vs HCB, P = 0.0053; **Figure 4D**) were decreased in the CB groups compared with the CS group significantly. The changes in protein expression levels of Occludin, Claudin-1 and Claudin-4 were probably coincided with those of mRNA expression. However, there were no significant differences in ZO-1 protein expression among any of the groups (P >0.05) (**Figures 4F, G**), which showed the discordant mRNA and protein expression. Moreover, the discordant mRNA and protein expression was also found in Occludin level of LCB group. Furthermore, we investigated the effects of CB RH2 on the MUC-2 mRNA expression. Our data showed that the gene expression level of MUC-2 was decreased in the CS group compared to the control group (P = 0.05) and CB RH2 administration enhanced mucin production (CS vs LCB, P = 0.0414; CS vs HCB, P = 0.0139; **Figure 4E**).

## Effects of CB RH2 on the Mucosal Immune Barrier and Immune System Under Ceftriaxone-Induced Dysbiosis

**Figure 5:**
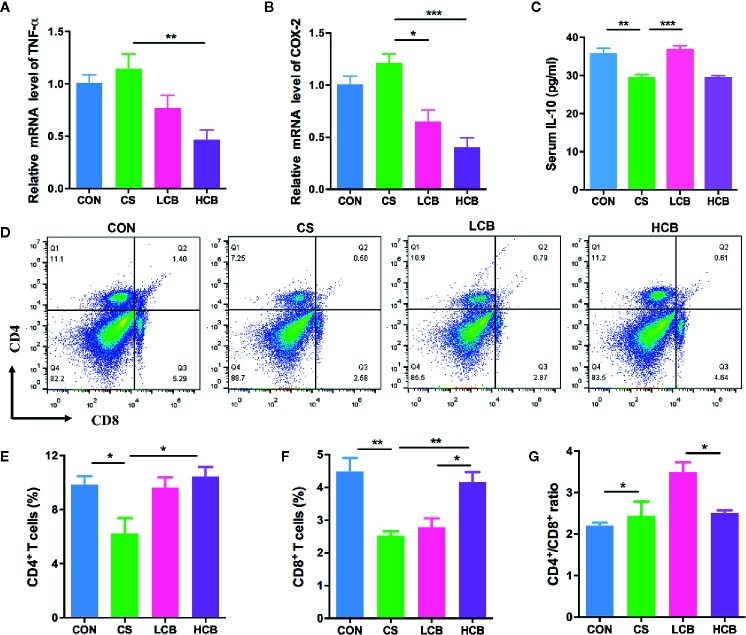
Anti-inflammatory effect of CB RH2 on mice with ceftriaxone-induced intestinal dysbacteriosis. **(A, B)** The relative RNA expression of genes encoding tumor necrosis factor alpha (TNF-α) and cyclooxygenase-2 (COX-2) in colon tissues of mice (n = 5); **(C)** IL-10 of serum were detected by ELISA following the manufacturer’s protocol (n = 5); **(D)** Representative flow cytometry plots of CD4^+^ and CD8^+^ T cells identified (n = 3); **(E, F)** Percentage of CD4^+^ and CD8^+^ T cells in spleen; **(G)** The proportion of CD4^+^/CD8^+^. All data were evaluated as mean ± SEM, *p <0.05, **p <0.01, ***p <0.001.

To address whether supplementation of CB RH2 could suppress colon inflammation, we investigate the level of mRNA expression of proinflammatory factors, including tumor necrosis factor alpha (TNF-α) and cyclooxygenase-2 (COX-2). We found that CB RH2 administration significantly reduced the expression of TNF-α (CS vs HCB, P = 0.0061; **Figure 5A**) and COX-2 (CS vs LCB, P = 0.0108; CS vs HCB, P = 0.0008; **Figure 5B**). To determine the anti-inflammatory effect of CB RH2, the serum levels of interleukin-10 (IL-10) was measured. Low concentration of CB RH2 treatment promoted IL-10 cytokine production (P = 0.0007; **Figure 5C**). However, high concentration treatment had no effects on serum IL-10. Subsequently, the T cells in the spleen including helper T cells (CD3^+^CD4^+^ T cells) and cytotoxic T cells (CD3^+^CD8^+^ T cells) were further investigated. The results revealed that the proportions of CD3^+^CD4^+^ T cells (P = 0.028; **Figures 5D, E**) and CD3^+^CD8^+^ T cells (P = 0.0086; **Figures 5D, F**) in the HCB group were observably upregulated in comparison to the CS group. However, CB RH2 could not reverse the higher ratio of CD4^+^/CD8^+^ in CS treated mice (**Figure 5G**). Taken together, ceftriaxone administration caused gut inflammation and CB RH2 may have an effect on epithelial cell tight junctions and may reduce inflammation in colonic tissue.

### CB RH2 Promotes Expansion of CD4^+^ T Cells Obtained From PPs *In Vitro*

**Figure 6:**
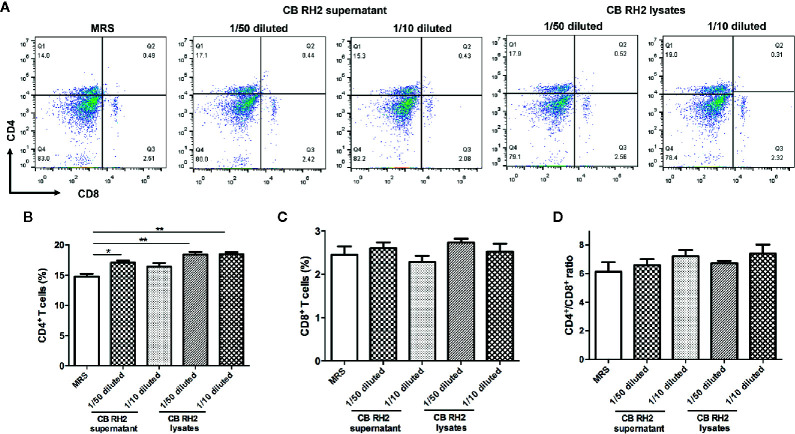
CB RH2 and its metabolites promote expansion of CD4^+^ T cells. **(A)** Representative flow cytometry plots; **(B, C)** Percentage of CD4^+^ and CD8^+^ T cells in Payer’s patches (PPs) of mice post stimulation of the supernatant and lysate of CB RH2; **(D)** The proportion of CD4^+^/CD8^+^. The MRS medium was used as control. All data were evaluated as mean ± SEM (n = 3), *p <0.05, **p <0.01.

PP-cells provide the most important defense mechanism for intestinal immunity. To determine whether protective effects of CB RH2 were directly associated with the upregulation of T cells, we further isolated the lymphocytes from the PPs of control mice and incubated them with the supernatant and lysate of CB RH2. We found that the percentages of CD4^+^ T cells in PPs were significantly higher following stimulation with both the supernatant (P = 0.03) and lysate (P <0.01) of CB RH2, whereas there was no effect on CD8^+^ T cells and the ratio of CD4^+^/CD8^+^ (**Figure 6**). This suggested that both of CB RH2 and its metabolites had direct effects on CD4^+^ T cells, but not CD8^+^ T cells or CD4^+^/CD8^+^ ratio in PPs.

## Discussion

Extensive evidence indicates that treatment with antibiotics has significant effects on the structure of the intestinal microbiota. As a clinical commonly used antibiotic, ceftriaxone can damage the intestinal epithelium barrier and disrupt the equilibrium of intestinal flora (Li et al., 2015). Recently, the effects of probiotics supplement on gut dysbiosis induced by antibiotics have been extensively studied, which can help to improve gastrointestinal barrier function, and affect both the mucosal and systemic immune systems (Marzorati et al., 2020). In the current study, we observed that probiotic CB RH2 supplementation improved intestinal mucosal inflammation and epithelial damage by dysbiosis of administration of ceftriaxone, and then *Bacteroides*, *Oscillibacter*, *Desulfovibrio*, *Mucispirillum* and *Parabacteroides* were predominantly changed.

To determine whether supplementation of CB RH2 has an immunomodulatory and metabolic role in regulating gut homeostasis, we administered ceftriaxone to BALB/C mice for 7 days to induce dysbiosis (**Figure 1A**). We confirmed that CB RH2 could significantly reduce mortality; thereby promote the recovery from ceftriaxone-induced dysbiosis (**Figure 1B**). Macroscopic findings revealed no significant changes in the length of colon even after ceftriaxone administration (**Figure 1C**). We predicted the CS group to show shorter colon compared with other three groups. However, this study showed the different result with our previous study with colitis model induced by DSS (Li et al., 2019). Similarly, conflicting results were also found in other studies (Hayashi et al., 2013; Hagihara et al., 2020), whereas the reason was unclear.

Similar to previous work, our studies have revealed that antibiotics induce gut dysbiosis. In this study, OTUs number in four groups showed no significant differences but high concentration of CB RH2 changed α-diversity and β-diversity of gut microbiota in ceftriaxone-treated mice (**Figure 2**). We analyzed the specific changes in the gut microbiota at the phylum and genus levels. The gut microbiota predominantly composed of four phyla: *Firmicutes*, *Bacteroidetes*, *Proteobacteria* and *Actinobacteria*. The increased *Firmicutes*/*Bacteroidetes* (F/B) abundance ratio was seen in CS group, which was in line with previous studies (Kong et al., 2020; Liu et al., 2020b). After the CB RH2 administration, especially in low dose, *Bacteroidetes* was significantly increased and F/B ratio was significantly decreased relative to the CS group. In a study of a rat model of severe acute pancreatitis with intra-abdominal hypertension, Zhao et al. (2020) reported that *C. butyricum* or butyrate could increase the relative abundance of *Bacteroidetes* significantly compare with both of sham and model groups. It was also observed that the proportion of *Deferribacteres* was significantly decreased following high concentration of CB RH2 treatment compared with CS group. The abundance of *Deferribacteres* has been previously observed in association with exacerbated intestinal inflammation in previous studies of colitis model (Selvanantham et al., 2016). The analysis in genus level showed application of CB RH2 decreased the proportion of *Oscillibacter*, *Desulfovibrio*, *Mucispirillum* and *Parabacteroides* (**Figure 2L**). The genus *Oscillibacter* was found in human gut microbiota related to a disease or pathologic state (Mondot et al., 2011; Lam et al., 2012). Upregulated abundance of *Oscillibacter* had been found in stroke and transient ischemic attack patients and closely related to gut permeability and host inflammation (Yin et al., 2015). *Desulfovibrio*, an endotoxins (such as LPS) producer, were thought to be positively associated with intestinal inflammation (Liu B. et al., 2020). Our previous study showed that a probiotic-enriched dietary intervention could decrease the abundance of *Desulfovibrio* in high-fat diet-induced obesity in rats (Li et al., 2020). It could be decreased by *C. butyricum* or butyrate in the study of Zhao et al. (2020). As part of the phylum *Deferribacteres*, *Mucispirillum* is a core member of murine gut microbiota, which can colonize the intestinal tract from the stomach to the colon (Xiao et al., 2019). It has been shown to be positively associated with pro-inflammatory MCP-1 (Shintouo et al., 2020). The represented species, namely, *Mucispirillum schaedleri*, was considered a pathobiont that was increased in DSS-induced colitis (Rooks et al., 2014). Similar as *Mucispirillum*, *Parabacteroides* was also positively associated with pro-inflammatory MCP-1 (Shintouo et al., 2020). A recent study showed that *Parabacteroides* was increased in a zoonotic parasitic disease, which could raise the risk of infections (Hu et al., 2020). These results suggested that CB RH2 might be involved in regulation of the intestinal microbiota by decreasing the pro-inflammatory bacteria (*Deferribacteres*, *Oscillibacter*, *Desulfovibrio*, *Mucispirillum* and *Parabacteroides*). It’s worth noting that administration of CB RH2 in high dose promoted the relative abundance of Lactobacillus (P = 0.052, **Figure 2K**), which were thought to be beneficial to several digestive diseases including IBS and IBD. This result was consistent with other studies (Liu et al., 2020b; Zhao et al., 2020).

Intestinal barrier integrity plays a fundamental role in healthy gut function. The intestinal barrier disruptions that facilitate the uptake of harmful agents are often associated with the alterations of gut microbiome (Grosheva et al., 2020). In current study, histological and ultrastructural abnormalities were detected in the colon of mice treated with ceftriaxone, which were reversed by CB RH2 intervention (**Figure 3**). Moreover, CB groups showed decreased gut permeability, which was indicated by the demotion of endotoxins such as LPS in the serum. As previous studies reported that defensins could protect the integrity of the epithelial barrier (Han et al., 2015; Ou et al., 2020), we determined whether CB RH2 could upregulate the defensins levels. However, our results showed higher levels of defensins in CS group than other groups, which was consistent with our previous study with ceftriaxone-induced dysbiosis (Li et al., 2015). As described in several recent studies, defensins could also be detrimental to host defense by enhancing adhesion and invasion of certain enteric pathogens (Wilson et al., 2017; Xu et al., 2018). In addition, these increased small peptides may further develop their antimicrobial activity against intestinal microbiota and induce more serious dysbiosis. In the present study, the administration of CB RH2, especially in low dose, could downregulate α-defensin dramatically, which suggesting the intestinal barrier protection.

The tight junction (TJ), which controls the integrity and permeability of intestinal epithelium, is composed of the TJ proteins such as Occludin, zonula occludens-1 (ZO-1), Claudin-1 and Claudin-4 (Bai et al., 2020). Moreover, disruptions in the tight junction barrier lead to impair the gut epithelial barrier function, which are involved in the pathogenesis of many intestinal disorders (Binienda et al., 2020). We found that CB RH2 administration, especially in high dose, upregulated intestinal mRNA levels of Occludin and ZO-1 (**Figure 4**), which was consistent with previous studies (Xiao et al., 2018; Liu et al., 2020a). Interestingly, the present results suggested that the protein and mRNA expression levels of Claudin-1 and Claudin-4 were significantly decreased in the CB RH2-treated groups. The exact functions of Claudin-1 and Claudin-4 remain debated, with contradicting findings suggesting context-dependent functions and potential roles both as seal and pore formers. As a pore former, Claudin-2 was detected overexpressed in the pathological response, which was reversed by *C. butyricum* or butyrate (Zhao et al., 2020). Therefore, the pore-forming Claudin-1 and Claudin-4 proteins may also play the role in increasing paracellular permeability. For example, previous study has reported that Claudin-1 expressed in pathological conditions and increase brain endothelial barrier permeability by altering the interactions of other proteins within the TJ complex (Sladojevic et al., 2019). Both silencing and overexpression of Claudin-4 in tubular cell lines were reported to reduce Na^+^ permeability (Van Itallie and Anderson, 2006; Borovac et al., 2012). The mucus biofilm, which covers the intestinal epithelium and the tight junctions, protects the barrier integrity and function (Wallace et al., 2011). Evidence shows a decrease in the thickness of adherent mucus layer during ulcerative colitis that is linked to genetic changes of MUC-2, the most abundant mucin protein in the intestine (Formiga et al., 2020). Our results indicated that CB RH2 upregulated RNA expression level of MUC-2, which was downregulated by ceftriaxone treated, thereby promoting stronger epithelial barrier integrity.

The gut microbiome is antigen to the immune system and plays a vital role in the development and regulation of the immune system, which is responsible for fighting off infections. Proinflammatory cytokines are known to be mediators during the onset of disease, and they play critical roles in immune status and inflammatory response. Tumor necrosis factor alpha (TNF-α) is one of the major proinflammatory factors relevant to the pathogenesis of IBD (Schultheiss et al., 2019). Popivanova et al. (2008) reported that the combined treatment with Azoxymethane and DSS induced the intracolonic expression of TNF-α and blocking of TNF-α reversed carcinoma progression with less inflammatory infiltrate. Cyclooxygenase-2 (COX-2), an inducible enzyme, drives inflammation and has been found to be highly expressed in patients with IBD (Nair et al., 2011). Interleukin-10 (IL-10), a potent anti-inflammatory cytokine, is capable of suppressing a number of proinflammatory signals associated with intestinal inflammatory diseases, such as ulcerative colitis and Crohn’s disease (Fay et al., 2020). Previous studies have reported that CB administration reduced inflammatory cytokines, including IL-1β, IL-6, COX-2, and TNF-α, while increased IL-10 expression levels in colon tissue (Hagihara et al., 2020; Liu et al., 2020a). Consistent with these reports, our results exhibited that high concentration of CB RH2 decreased the level of proinflammatory cytokines TNF-α and the level of enzyme COX-2. Furthermore low concentration of CB RH2 promoted IL-10 production in serum. However, high concentration of CB RH2 had no effects on serum IL-10, which might be caused by some unknown factors. Besides, proinflammatory cytokines can directly or indirectly disrupt intestinal barrier function (Bruewer et al., 2003; Song et al., 2019). For example, it has been reported that TNF-α increases intestinal epithelial TJ permeability by modulating myosin light chain kinase (MLCK) promoter activity *via* the NF-κB signaling pathway in Caco‑2 cells (Ye et al., 2006).). The intestinal floras activate the T cells, which enter the blood circulation through lymph circulation, and then they are delivered to immune organs such as spleen (Breslin, 2014). We further examined the T lymphocyte subpopulations in ceftriaxone-induced dysbacteriosis mice. Consistent with a previous study (Guo et al., 2020), we found that significantly lower frequency of splenic CD4^+^ and CD8^+^ T cells, and significantly higher CD4^+^/CD8^+^ ratio were detected in CS group. Interestingly, treatment with CB RH2 in high dose significantly eliminated the ceftriaxone-mediated decrease in the frequency of splenic CD4^+^ and CD8^+^ T cells (**Figure 5**), which can help to detect and fight off infections (Eberl et al., 2015). Hagihara et al. (2020) also reported that CBM 588 administration resulted in elevation of CD4 cells. However, CB RH2 administration could not affect the CD4^+^/CD8^+^ ratio. Unchanged CD4^+^/CD8^+^ ratio and a significant increase in CD4^+^ and CD8^+^ T cells may indicate that the immune response of ceftriaxone-induced dysbacteriosis mice was maintained by maintaining normal CD4^+^/CD8^+^ ratio and by increasing CD4^+^ and CD8^+^ T cells. Therefore, the present study verified that CB RH2 could enhance T cells responses and inhibit intestinal inflammation in ceftriaxone-induced dysbacteriosis that associated with a reduction of proinflammatory cytokine expression and an increase in antiinflammatory cytokines. However, the mechanism of protection remains to be elucidated. As a result, we subsequently isolated PPs from mice and incubated it with CB RH2 supernatant and lysates, aiming to determine the direct effects on T cells responses. CB RH2 supernatant was regarded as bacterial secretion, while lysates were regarded as intracellular components of CB RH2. The results of *in vitro* showed that both of CB RH2 and its metabolites had direct effects on CD4^+^ T cells, but not CD8^+^ T cells or CD4^+^/CD8^+^ ratio in PPs (**Figure 6**). We speculated that the role of supernatant was closely associated with changes of pH during the growth of CB RH2, which produced acetic and butyric acid. It is noteworthy that the effect of CB RH2 lysates was stronger than that of supernatant, which indicated that the effect on CD4^+^ T cells was largely based on the intracellular components of CB RH2, such as intracellular proteins. However, few studies have focused on the role of CB intracellular components. Further research is needed to better understand the effects of CB RH2 on CD4^+^ T cells. CD4^+^ T cells can help detect and fight bacterial and viral infections. Our study was therefore consistent with a previous clinical study, which reported that supplement of *C. butyricum* and *Bifidobacterium* improved the balance of CD4^+^/CD8^+^ T cells by dramatically increasing the percentage of CD4^+^ T cells (Zhang et al., 2016).

In conclusion, our study suggested that CB RH2 changed the composition of gut microbiota in phylum and genus level, decreased the F/B ratio, and decreased the opportunistic pathogen in ceftriaxone-treated mice. Furthermore, CB RH2 could improve the intestinal health through improving the mucous layer and the tight junction barrier, and modulating the intestinal mucosal and systemic immune system. However, some limitations of this study should be noted. More optimized study design, a larger sample size, and different sampling times would benefit future studies. Further studies are required to fully understand the mechanisms and the effective components in CB RH2 against antibiotics, which would provide further evidence of the anti-inflammatory potentials of CB RH2 for ceftriaxone-induced dysbiosis.

## Data Availability Statement

The original contributions presented in the study are included in the article/supplementary material. Further inquiries can be directed to the corresponding authors.

## Ethics Statement

The animal study was reviewed and approved by the committee for animal care and use at Dalian Medical University (SYXK [Liao] 2018-0002).

## Author Contributions

YL performed the experiments, analyzed the data and wrote the manuscript. MaL, HL, XS, CL, YC, WY, BG and XW performed the experiments. YL and XW analyzed and interpreted the data. HC and LZ designed the research. MiL and JY obtained the funding, designed the research, and revised the manuscript. All authors contributed to the article and approved the submitted version.

## Funding

This study was supported by the National Natural Science Foundation of China (31900920), the Nature Science Foundation of Liaoning Province, China (2015020262 and 2019-ZD-0648), and the Dalian Science and Technology Innovation Project (2020JJ27SN068). This work was also supported by Liaoning Provincial Program for Top Discipline of Basic Medical Sciences, China.

## Conflict of Interest

Authors HC and LZ were employed by the company Hangzhou Grand Biologic Pharmaceutical INC.

The remaining authors declare that the research was conducted in the absence of any commercial or financial relationships that could be construed as a potential conflict of interest.
